# Supplementary figures and images for: Real-time profilometry by bicolor grating video projection
Source: PLoS One. 2021 Nov 29;16(11):e0259569. doi: 10.1371/journal.pone.0259569 (PMC8629212; doi:10.1371/journal.pone.0259569)

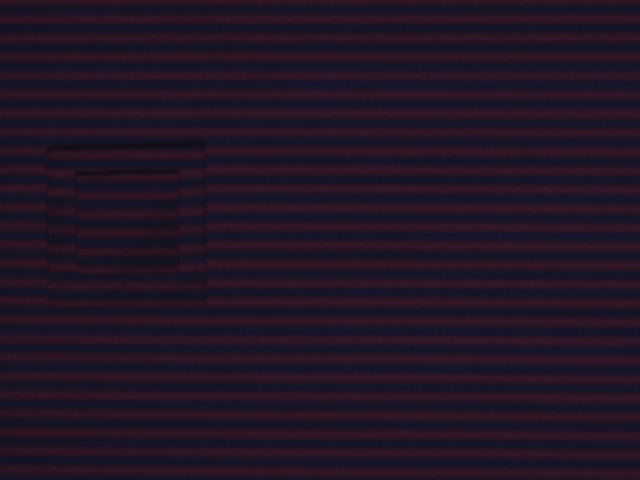

Supplement: S1 Visualization — The 12 frames of deformed patterns collected during the movement of the double-layer rectangular model are extracted and made into a gif. (GIF) [file pone.0259569.s001.Gif]

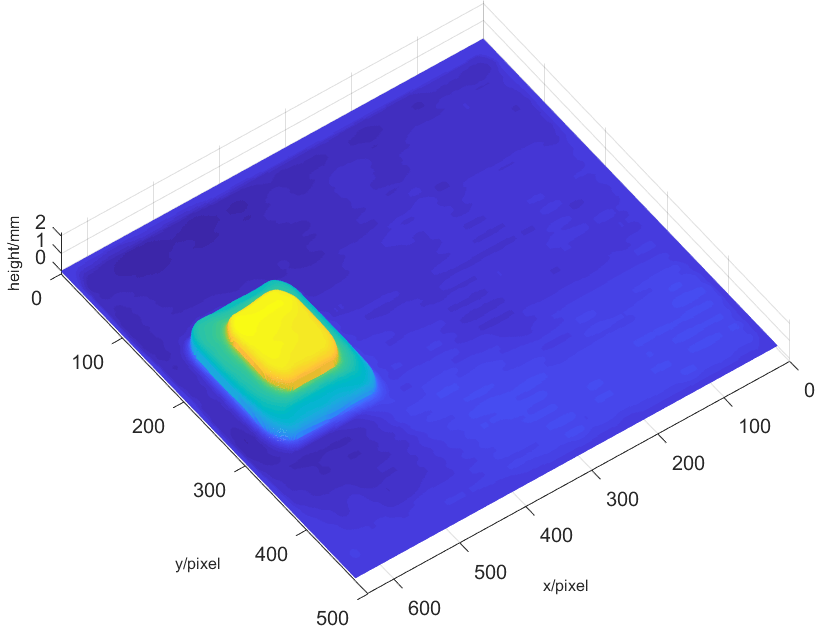

Supplement: S2 Visualization — The 12 frames of the 3D shapes reconstructed by the real-time online moving double-layer rectangular model under the corresponding moving states and made into a gif. (GIF) [file pone.0259569.s002.Gif]

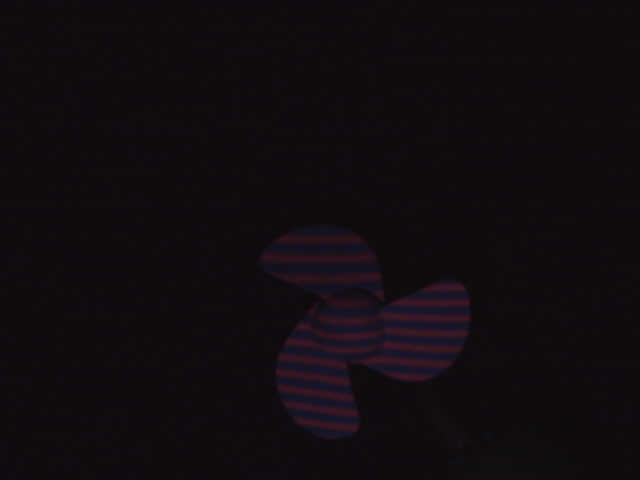

Supplement: S3 Visualization — The 10 frames of deformed patterns collected during the movement of the sector model are extracted and made into a gif. (GIF) [file pone.0259569.s003.Gif]

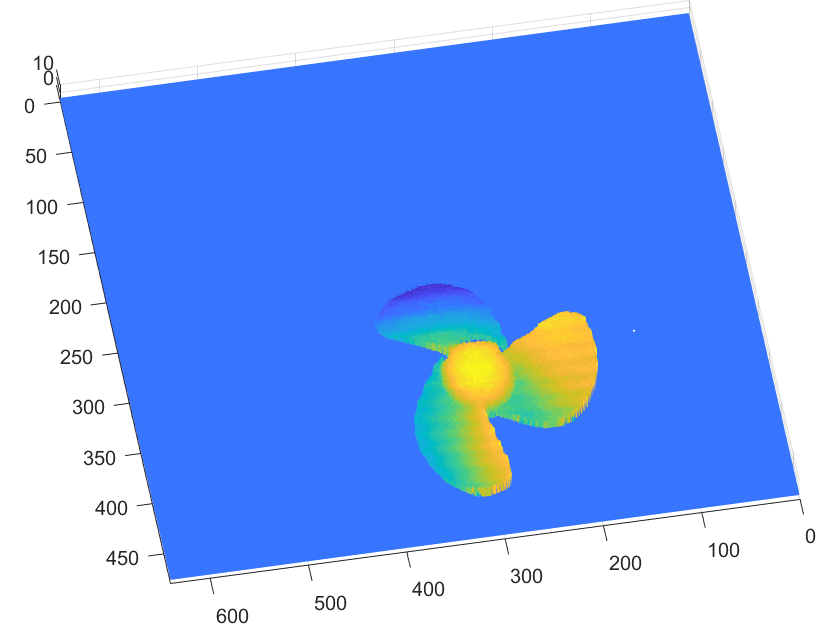

Supplement: S4 Visualization — The 10 frames of the 3D shapes reconstructed by the real-time online moving sector model under the corresponding moving states and made into a gif. (GIF) [file pone.0259569.s004.Gif]
