# Supplementary material for: Real-time profilometry by bicolor grating video projection
Source: PLoS One. 2021 Nov 29;16(11):e0259569. doi: 10.1371/journal.pone.0259569 (PMC8629212; doi:10.1371/journal.pone.0259569)
Supplement: Attachment — Submitted filename: Response to Reviewers (10).docx [file pone.0259569.s005.docx]

Dear Editor and Reviewers:

Thank you very much for your excellent editing work of our manuscript entitled “Real-time profilometry by bicolor grating video projection ([PONE-D-21-03909]-[EMID:a2a144e40538be21])”. And we also appreciate the reviewers for their valuable comments about our manuscript. Those comments are all valuable and very helpful for revising and improving our manuscript. We have studied the comments carefully and have made the correction which we hope to be able to meet the approval. The Response to Reviewer and Editor Comments is attached below. Hope our revised manuscript meets the high publication standard set by PLOS ONE.

Sincerely,

Yujiao Zhang, Yiping Cao, Haitao Wu, Haihua An, Xiuzhang Huang Sichuan University

Reviewer 1:

Comments to the Author

This paper proposed a novel real-time 2+1 three-dimensional measuring method based on a two-color fringe projection. In general, the structure of this paper is clear, and there are some new ideas especially on the fusion and phase recovery of two-color fringes. But the article still has many problems before published.

1. The English writing needs to be revised, because there are too many wording mistakes which influence the readability of the article. For example, in line 40, ‘faster speed’ should be placed by ‘higher speed’; in line 41, ‘wider measurement application range’, the meaning is not clear; in line 48, ‘application scope is very narrow’; many wrong uses of ‘Figs’ in line 170…; in line 174 wrong use of ‘quasi-true’; in line 222, wrong use of ‘cow’; invalid format in line 170 ‘π/2’, etc.

Response:

Thank you for raising this question, and I am sorry that my English grammatical errors and improper expressions have caused you difficulties in reading. I have corrected the grammar of the thesis from beginning to end. After receiving the modification comments, I did the real-time experiment again. And this time, I selected the 250th column of the reconstructed heart, so I changed the corresponding part of the experiment expression accordingly.

Revision:

1) In page 2 line 38, “slower speed” has been revised as “a lower speed”.
2) In page 2 line 38, “higher measuring accuracy, wider measurable range” has been revised as “a higher measuring accuracy, a wider range of heights of measuring object”.
3) In page 2 line 41, “faster measurement speed, higher measuring accuracy, and wider measurement application range” has been revised as “a higher measuring speed, higher measuring accuracy, a wider measuring application range”.
4) In page 9 line 172, “Figs 2(c) and 2(d)” has been revised as “Fig 2(c) and Fig 2(d)”.
5) In page 9 line 173, “Figs 2(e) and 2(f)” has been revised as “Fig 2(e) and Fig 2(f)”.
6) In page 9 line 174, “Figs 2(g) and 2(h)” has been revised as “Fig 2(g) and Fig 2(h)”.
7) In page 10 line 181, “Figs 3(c)-(e)” has been revised as “Fig 3(c)–Fig3 (e)”.
8) In page 12 line 208, “Figs 4 (a)-(c)” has been revised as “Fig 4(a)-Fig 4(c)”.
9) In page 12 line 209, “Figs 4(d)-(f)” has been revised as “Fig 4(d)-Fig 4 (f)”.
10) In page 12 line 211, “Figs 4(g)-(i)” has been revised as “Fig 4(g)-Fig 4(i)”.
11) In page 14 line 231, “Figs 5(b) and 5(d)” has been revised as “Fig 5(b) and Fig 5(d)”.
12) In page 9 line 176, page 12 line 216, page 13 line 220 “quasi-true value” has been revised as “quasi-truth”.
13) In page 13 between line 223 and 224, “**323th cow**” has been revised as “**250th column**”.
14) In page 14 line 228, “323th cow” has been revised as “250th column”.
15) In page 7 line 131, page 9 line 172 “π/2” has been revised as “”.
16) In page 3 line 61, “is” has been revised as “was”.
17) In page 3 line 61, “Firstly” has been revised as “First”.
18) In page 3 line 62, “are” has been revised as “were”.
19) In page 3 line 63, “is” has been revised as “was”.
20) In page 3 line 64, “containing” has been revised as “that covered”.
21) In page 3 line 65, “can” has been revised as “could”.
22) In page 3 line 66, “measured object” has been revised as “object measured”.
23) In page 4 line 73, page 4 line 74, page 4 line 75, page 5 line 104, page 5 line 105, page 5 line 106, page 5 line 107, page 6 line 115, “is” has been revised as “expresses”.
24) In page 6 line 114, “are” has been revised as “denote”.
25) In page 4 line 86, “expressions (2) and (3) and (4)” has been revised as “Eq (2), Eq (3) and Eq (4)”.
26) In page 6 line 114, we add two words “the”.
2. There are many descriptions hard to understand. For example, line 33-36; line 38-40; line 97-99; line 123-125…

Response:

I am sorry that I did not express clearly enough in these places, which caused your reading difficulties. I re-expressed the content of these places.

Revision:

1) In page 2 line 32-36, The content “FTP requires only one frame of phase shift diagram, retains the fringe frequency in the frequency domain, removes high frequency noise and carrier waves, recovers the fringe pattern from the frequency domain to the spatial domain through inverse Fourier transform, reproduces the fringe field distribution, and finally calculates the phase value of the fringe field [13-14].” has been revised as “FTP requires only one frame of phase shift diagram, It is capable of retaining the fringe frequency in the frequency domain, removing high-frequency noise and carrier waves, recovering the fringe pattern from the frequency domain to the spatial domain via the inverse Fourier transform, reproducing the fringe field distribution, and lastly calculating the phase value of the fringe field [13-14]”.
2) In page 2 line 37-42, The content “Compared with FTP, PMP has slower speed but higher measuring accuracy, wider measurable range, less influence by different reflectivity on the surface of the object, and less influence by drastic changes or fractures on the surface of the object[15-16]. Modern 3D measurement is devoted to the research of faster measurement speed, higher measuring accuracy, and wider measurement application range, so PMP is a better choice[17-21] .” has been revised as “As opposed to FTP, PMP exhibits a lower speed but a higher measuring accuracy, a wider measurable range, less effect by different reflectivity values on object surface, and less effect exerted by drastic variations or fractures on object surface[15-16]. The modern 3D measurement is committed to investigating a higher measuring speed, higher measuring accuracy, as well as a wider measurement application range; thus, PMP is recognized as a more effective choice [17-21].”.
3) In page 3 line 48-49, I corrected the sentence “The technical conditions of the proposed method are limited and the application scope is very narrow[24]”to “The proposed algorithm has the limited technical conditions and a narrow application scope [24]”.
4) In page 3 line 55-57, this sentence is a bit difficult to read, so I corrected “Because the sensitivity of the uniform plane graph to motion blur is much lower than that of the fringe graph, moreover, compared with the traditional 2+1 phase shift algorithm, this method uses less one frame of image and has faster acquisition speed.” to “Since the sensitivity of the uniform plane graph to motion blur is significantly lower than that of the fringe graph, this method, compared with the conventional 2+1 phase shift algorithm, employs less one frame of image and exhibits a higher acquisition speed.”
5) In page 5 line 96-99, The content “If two frames of red and blue uniform background light with the same value as the DC component of the sinusoidal fringe are separately projected to solve the chromaticity imbalance problem caused by the different sensitivity of the CMOS camera to red and blue, then the red color of the same color image in the actual experiment is ignored..” has been revised as “If two frames of red and blue uniform background lights with the identical value as the DC component of the sinusoidal fringe are separately projected to address the chromaticity imbalance problem attributed to the different sensitivities of the CMOS camera to red and blue. Subsequently, the red color of the identical color image in the practical experiment is ignored. To possibly eliminate the chromaticity imbalance attributed the different sensitivities of the CMOS camera to the red and blue lights, two bicolor gratings are designed in advance:”.
6) In page 6 line 124-127, The content “Further analysis shows that $R_{r}(\text{x},y)$ and $R_{\text{b}}(x,y)$ have similar characteristics. It can be seen that the ratio of reflectivity of red channel and blue channel is in constant characteristic. This is caused by the chromaticity imbalance caused by the different sensitivities of CMOS camera to red and blue light.” has been revised as “As indicated from the in-depth analysis, $R_{r}(\text{x},y)$ and $R_{\text{b}}(x,y)$ exhibit the similar characteristics. which demonstrates that the ratio of reflectivity of the red channel and the blue channel exhibits the constant characteristic. This is attributed to the chromaticity imbalance attributed to the different sensitivities of the CMOS camera to the red and blue lights.”.
3. Many references are miscoded. For example, line 31, 36, 42…

Response:

Thank you so much for your question, and I am sorry that my carelessness caused this format problem. I have corrected the reference number hyperlink problem.

Revision:

There are three numbering errors in the preview state.

1) In page 2 line 31, Number “[7-8]” has updated the number hyperlink；
2) In page 2 line 36, Number “[13-14]” has updated the number hyperlink；
3) In page 2 line 42, Number “[17-21]” has updated the number hyperlink；
4. Suggest that the red number should be placed in the Fig.1.

Response:

Thank you very much for your valuable comments. After my consideration, I also think that your suggestions for modification are better.

Revision:

So I replace the Fig.1

**Fig.1 Experimental system**

Reference plane

Measured object

Stepping motor

DFM 72AUCO2

PLED-W20

between lines 155 and 156 in page 8 with the following figure

**Fig.1 Experimental system**

Reference plane

Measured object

Stepping motor

DFM 72AUCO2

PLED-W200

5. About the experiment, there was no quantitative analysis in the comparative experiment. It will be better to give the residual figure between each of the method and measurement accuracy values.

Response:

Thank you so much for your suggestion. I am sorry that I did not do the quantitative analysis of the experiment. Now I am supplementing the quantitative analysis of the experiment. Compared with the eight-step PMP, ZHANG S and the proposed method are compared to obtain the height error distribution and calculate the height root mean square error. We did a set of static experiments again We respectively added the height error distribution of the ZHANG S algorithm (Fig3 (f)) and the proposed method relative to the eight-step PMP (Fig3 (g)) to Fig.3 in the static face model measurement. Then, we separately calculated the height root mean square error relative to the eight-step PMP, the result of the algorithm of ZHANG S can be obtained as 0.1913, the proposed method can be obtained as 0.0629.

In the same way, we also did a set of real-time experiments again, and the cross-sectional view is replaced with the 250th column. We respectively added the height error distribution of the ZHANG S algorithm (Fig.5(e)) and the proposed method relative to the eight-step PMP(Fig.5(f)) to Fig.5 in the real-time heart model measurement.

Revision:

In page 10, between line 177 and line 178, the measurement results of the face model at rest.

B

A

**Fig.3 Experimental results and height error distribution.** (a)Object reconstructed by ZHANG S′s algorithm, (b)Object reconstructed by proposed algorithm, (c)Sectional view of the 3D profile of the object, (d)Magnified view of the area A

(e)Magnified view of area B, (f) Height error distribution of ZHANG S′s algorithm, (g) Height error distribution of proposed algorithm

(a)

(b)

(c)

(d)

(e)

(f)

(g)

The content of the analysis is added between line 187 and 192 in page 11, the content presents “As opposed to the results obtained with the eight-step PMP results, the algorithm height error of ZHANG S as shown in Fig 3(f), and the algorithm height error of the proposed method is shown in Fig 3(g). It can be clearly seen that the height error of the proposed method is smaller. Calculating the height root mean square error of the two methods, the result of the algorithm of ZHANG S can be obtained as 0.1913, the proposed method can be obtained as 0.0629.”.

In page 13, between line 223 and line 224, the measurement results of the heart model at real time.

(a)

(b)

(c)

**Fig.5 The 250th column comparison results of the reconstructed object.** (a) Sectional view of the 3D profile of the object, (b) Magnified view of the A area, (c) Magnified view of the B area(d) Magnified view of the C area, (e) Height error distribution of ZHANG S′s algorithm, (f) Height error distribution of proposed algorithm

A

C

B

(d)

(e)

(f)

The content of the analysis is added between line 235 and 239 in page 14, the content presents “As opposed to the results obtained with the eight-step PMP results, the real-time algorithm height error of ZHANG S as shown in Fig 5(e), and the real-time algorithm height error of the proposed method is shown in Fig 5(f). It can be clearly seen that the height error of the proposed method is smaller. Calculating the height root mean square error of the two methods, the result of the algorithm of ZHANG S can be obtained as 0.1546, the proposed method can be obtained as 0.0513.”.

6. I still have a question why the eight-step PMP was used for a reference not the five-step or the other method?

Response:

Thank you so much for asking this question, and I am sorry for not being able to describe in detail the advantages of 8-step PMP as a quasi-truth method in my article. The reason for using this method is that I read a thesis called “Gamma model and its analysis for phase measuring profilometry”. From the thesis I know that Phase measuring profilometry is a method of structured light illumination whose three-dimensional reconstructions are susceptible to error from nonunitary gamma in the associated optical devices. The effects of this distortion diminish with an increasing number of employed phase-shifted patterns, so the more PMP steps, the better the measurement effect. I added this thesis as number 30 to the reference, located at line 175, and added “30.	Liu, Kai, et al. "Gamma model and its analysis for phase measuring profilometry." JOSA A 27.3 (2010): 553-562.” in page 19 line 333-334.

7. High speed measurements are expected to give speed values and comparisons.

Response:

Thank you very much for your valuable suggestion. By controlling the speed of the stepper motor to control the real-time movement speed of the cardioid model, the corresponding root-mean-square is calculated according to the same method as the previous real-time comparison. The speed range of the stepper motor is 0~40mm/s. We choose 24mm/s, 30mm/s, and 36mm/s respectively for experimen

Revision:

The height root mean square is made into a table and the results are as follows and added the table and analysis content to page 15 between line 246 and 252:

Speed(mm/s)

Table.1 The height root mean square error at different speeds

method

|  | 24mm/s | 30mm/s | 36mm/s |
| --- | --- | --- | --- |
| ZHANG S | 0.1435 | 0.1546 | 0.1866 |
| Propose | 0.0442 | 0.0513 | 0.0753 |

In page 14 line 240-242, we added the sentence of “According to the above comparison method, we select the speed as 24ms/s, 30ms/s, and 36ms/s respectively by controlling the speed of the stepper motor to complete real-time experiment. Then, to calculate the corresponding root mean square value and make a table1 expressed below:”

In page 15 line 244-246, we added the sentence of “It can be observed that the measuring effect becomes worse as the speed increases. The reason for this result is that the higher the speed of the object was, the greater the amplitude of the object’s vibration would be, the more factors that the captured image would interfere with, the more unstable resulting distorted image will be and the worse the measurement effect would be.”

Reviewer #2: The experimental section does not specify if the data is cherry-picked or averaged or repeatable over several frames. Unfortunately, the manuscript contains lots of English errors, and also, references are displayed as "Error! Reference source not found."

1. The experimental section does not specify if the data is cherry-picked or averaged or repeatable over several frames.

Response:

Thank you for raising this question, and sorry for not being able to express it clearly. This article uses video raster projection, and what the camera captures is also a video image. I selected three sets of adjacent two frames of images for three-dimensional measurement. For example, the video images selected in this paper have a total time of 9s, which corresponds to more than 160 frames of images. I selected frames 29-30, 89-90, and 139-140 to complete the three-dimensional measurement.

2. Unfortunately, the manuscript contains lots of English errors.

Response:

Thank you for raising this question, and I am sorry that my English grammatical errors and improper expressions have caused you difficulties in reading. I have corrected the grammar of the thesis from beginning to end.

Revision:

1) In page 2 line 38, “slower speed” has been revised as “a lower speed”.
2) In page 2 line 38, “higher measuring accuracy, wider measurable range” has been revised as “a higher measuring accuracy, a wider range of heights of measuring object”.
3) In page 2 line 41, “faster measurement speed, higher measuring accuracy, and wider measurement application range” has been revised as “a higher measuring speed, higher measuring accuracy, a wider measuring application range”.
4) In page 9 line 172, “Figs 2(c) and 2(d)” has been revised as “Fig 2(c) and Fig 2(d)”.
5) In page 9 line 173, “Figs 2(e) and 2(f)” has been revised as “Fig 2(e) and Fig 2(f)”.
6) In page 9 line 174, “Figs 2(g) and 2(h)” has been revised as “Fig 2(g) and Fig 2(h)”.
7) In page 10 line 181, “Figs 3(c)-(e)” has been revised as “Fig 3(c)–Fig3 (e)”.
8) In page 12 line 208, “Figs 4 (a)-(c)” has been revised as “Fig 4(a)-Fig 4(c)”.
9) In page 12 line 209, “Figs 4(d)-(f)” has been revised as “Fig 4(d)-Fig 4 (f)”.
10) In page 12 line 211, “Figs 4(g)-(i)” has been revised as “Fig 4(g)-Fig 4(i)”.
11) In page 14 line 231, “Figs 5(b) and 5(d)” has been revised as “Fig 5(b) and Fig 5(d)”.
12) In page 9 line 176, page 12 line 216, page 13 line 220 “quasi-true value” has been revised as “quasi-truth”.
13) In page 13 between line 223 and 224, “**323th cow**” has been revised as “**250th column**”.
14) In page 14 line 228, “323th cow” has been revised as “250th column”.
15) In page 7 line 131, page 9 line 172 “π/2” has been revised as “”.
16) In page 3 line 61, “is” has been revised as “was”.
17) In page 3 line 61, “Firstly” has been revised as “First”.
18) In page 3 line 62, “are” has been revised as “were”.
19) In page 3 line 63, “is” has been revised as “was”.
20) In page 3 line 64, “containing” has been revised as “that covered”.
21) In page 3 line 65, “can” has been revised as “could”.
22) In page 3 line 66, “measured object” has been revised as “object measured”.
23) In page 4 line 73, page 4 line 74, page 4 line 75, page 5 line 104, page 5 line 105, page 5 line 106, page 5 line 107, page 6 line 115, “is” has been revised as “expresses”.
24) In page 6 line 114, “are” has been revised as “denote”.
25) In page 4 line 86, “expressions (2) and (3) and (4)” has been revised as “Eq (2), Eq (3) and Eq (4)”.
26) In page 6 line 114, we add two words “the”.
27) In page 2 line 32-36, The content “FTP requires only one frame of phase shift diagram, retains the fringe frequency in the frequency domain, removes high frequency noise and carrier waves, recovers the fringe pattern from the frequency domain to the spatial domain through inverse Fourier transform, reproduces the fringe field distribution, and finally calculates the phase value of the fringe field [13-14].” has been revised as “FTP requires only one frame of phase shift diagram, It is capable of retaining the fringe frequency in the frequency domain, removing high-frequency noise and carrier waves, recovering the fringe pattern from the frequency domain to the spatial domain via the inverse Fourier transform, reproducing the fringe field distribution, and lastly calculating the phase value of the fringe field [13-14]”.
28) In page 2 line 37-42, The content “Compared with FTP, PMP has slower speed but higher measuring accuracy, wider measurable range, less influence by different reflectivity on the surface of the object, and less influence by drastic changes or fractures on the surface of the object[15-16]. Modern 3D measurement is devoted to the research of faster measurement speed, higher measuring accuracy, and wider measurement application range, so PMP is a better choice[17-21] .” has been revised as “As opposed to FTP, PMP exhibits a lower speed but a higher measuring accuracy, a wider measurable range, less effect by different reflectivity values on object surface, and less effect exerted by drastic variations or fractures on object surface[15-16]. The modern 3D measurement is committed to investigating a higher measuring speed, higher measuring accuracy, as well as a wider measurement application range; thus, PMP is recognized as a more effective choice [17-21].”.
29) In page 3 line 48-49, I corrected the sentence “The technical conditions of the proposed method are limited and the application scope is very narrow[24]”to “The proposed algorithm has the limited technical conditions and a narrow application scope [24]”.
30) In page 3 line 55-57, this sentence is a bit difficult to read, so I corrected “Because the sensitivity of the uniform plane graph to motion blur is much lower than that of the fringe graph, moreover, compared with the traditional 2+1 phase shift algorithm, this method uses less one frame of image and has faster acquisition speed.” to “Since the sensitivity of the uniform plane graph to motion blur is significantly lower than that of the fringe graph, this method, compared with the conventional 2+1 phase shift algorithm, employs less one frame of image and exhibits a higher acquisition speed.”
31) In page 5 line 96-99, The content “If two frames of red and blue uniform background light with the same value as the DC component of the sinusoidal fringe are separately projected to solve the chromaticity imbalance problem caused by the different sensitivity of the CMOS camera to red and blue, then the red color of the same color image in the actual experiment is ignored..” has been revised as “If two frames of red and blue uniform background lights with the identical value as the DC component of the sinusoidal fringe are separately projected to address the chromaticity imbalance problem attributed to the different sensitivities of the CMOS camera to red and blue. Subsequently, the red color of the identical color image in the practical experiment is ignored. To possibly eliminate the chromaticity imbalance attributed the different sensitivities of the CMOS camera to the red and blue lights, two bicolor gratings are designed in advance:”.
32) In page 6 line 124-127, The content “Further analysis shows that $R_{r}(\text{x},y)$ and $R_{\text{b}}(x,y)$ have similar characteristics. It can be seen that the ratio of reflectivity of red channel and blue channel is in constant characteristic. This is caused by the chromaticity imbalance caused by the different sensitivities of CMOS camera to red and blue light.” has been revised as “As indicated from the in-depth analysis, $R_{r}(\text{x},y)$ and $R_{\text{b}}(x,y)$ exhibit the similar characteristics. which demonstrates that the ratio of reflectivity of the red channel and the blue channel exhibits the constant characteristic. This is attributed to the chromaticity imbalance attributed to the different sensitivities of the CMOS camera to the red and blue lights.”.
3. references are displayed as "Error! Reference source not found.

Response:

Thank you so much for your question, and I am sorry that my carelessness caused this format problem. I have corrected the reference number hyperlink problem.

Revision:

There are three numbering errors in the preview state.

1) In page 2 line 31, Number “[7-8]” has updated the number hyperlink；
2) In page 2 line 36, Number “[13-14]” has updated the number hyperlink；
3) In page 2 line 42, Number “[17-21]” has updated the number hyperlink；
