# Supplementary material for: Real-time profilometry by bicolor grating video projection
Source: PLoS One. 2021 Nov 29;16(11):e0259569. doi: 10.1371/journal.pone.0259569 (PMC8629212; doi:10.1371/journal.pone.0259569)
Supplement: Attachment — Submitted filename: Response to Reviewers.docx [file pone.0259569.s006.docx]

Dear Editor and Reviewers:

Thank you very much for your excellent editing work of our manuscript entitled “Real-time profilometry by bicolor grating video projection (PONE-D-21-03909R2- [EMID:ed7f0c6191b82490])”. And we also appreciate the reviewers for their valuable comments about our manuscript. Those comments are all valuable and very helpful for revising and improving our manuscript. We have studied the comments carefully and have made the correction which we hope to be able to meet the approval. The Response to Reviewer and Editor Comments is attached below. Hope our revised manuscript meets the high publication standard set by PLOS ONE.

Sincerely,

Yujiao Zhang, Yiping Cao, Haitao Wu, Haihua An, Xiuzhang Huang Sichuan University

Reviewer #2:

1. About the data - the authors have indicated in their response to my review that only three pairs of frames were used for the entire paper, out of 9 seconds of video with 160 frames. Some statistical error analysis of the other frames would be desirable, and the raw data should be made available as PLOS ONE's requirement is "PLOS journals require authors to make all data necessary to replicate their study's findings..."

Response:

I am sorry that I only provided three frames of data. In order to be able to provide real-time motion data more accurately, I kept the heart-shaped data and chose a new double-layer rectangular model for experimentation. The multi-frame deformed fringe information and real-time imaging information captured in the experiment are displayed in S1Visualization and S2 Visualizatio, respectively. To further prove the feasibility of real-time exercise, I used this method to measure more complex rotating sectors such as sectors. The multi-frame deformed fringe information and real-time imaging information captured in the experiment are displayed in S3 Visualization and S4 Visualization.

Revision:

1) In page 11 line 201, “heart” has been revised as “double-layer rectangular”.
   2) In page 11 line 203, “heart” has been revised as “double-layer rectangular”.
   3) In Fig4, “heart” has been revised as “double-layer rectangular”.
   4) In page 12 line 209, line 211, line 212, line 213, line 217 “heart” has been revised as “double-layer rectangular”.
   5) In page 13 line 226, “heart” has been revised as “double-layer rectangular”.
   6) In page 14 line 229, “heart” has been revised as “double-layer rectangular”.
   7) In page 12, between line 206 and line 207, I replaced the heart-shaped model in Fig.4 with a double-layer rectangular model

**Fig.4 Experimental results of real-time 3D measurement for moving double-layer rectangular model.** (a)-(c) 1^st^-3^rd^ deform patterns , (d)-(f) 1^st^-3^rd^ monochromatic deform pattens , (g)-(i)objects reconstructed of time 1 to 3

(a)

(b)

(c)

(d)

(e)

(f)

(g)

(h)

(i)

8) In page 13, between line 224 and line 225, I replaced the heart-shaped model in Fig.5 with a double-layer rectangular model.

(a)

(b)

(c)

A

C

(d)

B

**Fig.5 The 235th row comparison results of the reconstructed object. (a) Sectional view of the 3D profile of the object, (b) Magnified view of the A area, (c) Magnified view of the B area(d) Magnified view of the C area, (e) Height error distribution of ZHANG S′s algorithm, (f) Height error distribution of proposed algorithm**

(e)

(f)

9) In page 14 line 231-line233, “the results of the two real-time methods are close to the eight-step PMP, however, from Fig 5(b) and Fig 5(d),” has been revised as “the results of the proposed method are close to the eight-step PMP, ZHANG's method is not effective in restoring the middle connection of the double-layer rectangle. From Fig 5(b) and Fig 5(d)”.
10) In page 14 line 241, “0.1456” has been revised as “0.1055”.
11) In page 14 line 241, “0.0153” has been revised as “0.0157”.
12) In page 14, between line 244 and line 246, I replaced the new data in Table1 with a double-layer rectangular model.

Table.1 The height root mean square error at different speeds

method

Speed(mm/s)

|  | 24mm/s | 30mm/s | 36mm/s |
| --- | --- | --- | --- |
| ZHANG S | 0.0817 | 0.1055 | 0.1443 |
| Proposed method | 0.0410 | 0.0517 | 0.0706 |

13) In page 11, line 205, I add more deformed patterns with a double-layer rectangular model, and made a S1Visualization of them.
14) In page 12, line 213, I add more reconstructed results with a double-layer rectangular model, and made a S2 Visualization of them.
15) In page 15, line 253, I add more deformed patterns with a more complex sector model, and made a S3 Visualization of them.
16) In page 16, line 261, I add more reconstructed results with a more complex sector model, and made a S4 Visualization of them.
17) In page 15-16, between line 250 and line 263, I add the new reconstruction results with a more complex sector model.

In order to further prove the feasibility of this method, a more complex sector model was measured in real time according to the same method. The bicolor deformed patterns at different times are simultaneously collected by the CMOS camera, and each group of deformed patterns are captured at 30 frames per second (30fps), are shown in Visualization 3. The part of the experimental results is shown in Fig 6.

**Fig.6 Experimental results of real-time 3D measurement for moving sector model.** (a)-(c) 1^st^-3^rd^ deform patterns, (d)-(f) 1^st^-3^rd^ monochromatic deform pattens, (g)-(i)objects reconstructed of time 1 to 3

(a)

(b)

(c)

(d)

(e)

(f)

(g)

(h)

(i)

Fig 6(a)-Fig 6(c) respectively show the bicolor deformed patterns extracted by the real-time online moving sector model under the conditions of state 1, 2, and 3 respectively. Fig 6(d)-Fig 6 (f) respectively show the monochrome deformed patterns extracted by the real-time online moving sector model under the corresponding state conditions. Fig 6(g)-Fig 6(i) respectively show the 3D shapes reconstructed by the real-time online moving sector model under the corresponding time conditions, all reconstructed results are shown in Visualization 4. It can be observed that the 3D reconstruction of the sector model has also been well restored, and its rotating tense can be restored in real time, which further proves the real-time feasibility of the proposed method.

2. Unfortunately, the manuscript contains lots of English errors.

Response:

Thank you for raising this question, and I am sorry that my English grammatical errors and improper expressions have caused you difficulties in reading. I am very grateful for the grammatical errors you pointed out to me, and I corrected the corresponding content according to your suggestions.

Revision:

2) In page 1 line 20, “the proposed” has been revised as “the proposed method”.
   18) In page 2 line 32, “profilometry (FTP) [9-10]and” has been revised as “profilometry (FTP) [9-10] and”.
   19) In page 2 line 33, “, It” has been revised as “, it”.
   20) In page 2 line 36, “[13-14] PMP” has been revised as “[13-14]. PMP”.
   21) In page 3 line 51, “uniform plan” has been revised as “uniform plane illumination”.
   22) In page 4 line 87, “plane” has been revised as “camera”.
   23) In page 3 line 57, “less one frame of image” has been revised as “one frame less.
   24) In page 3 line 62-64, “First, only two frames of bicolor patterns substituting for the three frames of essential patterns in the traditional 2+1 3D real-time measuring method were encoded and arranged alternatively to synthesize into a repetitive bicolor grating video” has been revised as “First, only two frames of bicolor patterns are used, as against the three frames which are essential in the traditional 2+1 3D real-time measuring method. These two frames were encoded and arranged alternately to synthesize a repetitive bicolor grating video”.
   25) In page 3 line 64-65, “Then, the video was projected onto the object, the bicolor deformed patterns” has been revised as “Then the video was projected onto the object and the bicolor deformed patterns”.
   26) In page 5 line 99, “separately projected to address” has been revised as “separately projected, this can address”.
   27) In page 6 line 126, “which demonstrates that the ratio of reflectivity of the red channel and the blue channel exhibits the constant characteristic” has been revised as “The ratio of reflectivity of the red channel and the blue channel is a constant”.
   28) In page 7 line 153, “VeiwSonic” has been revised as “ViewSonic”.
   29) In page 7 line 154, “ImagingSource Company.” has been revised as “The Imaging Source”.
   30) In page 7 line 153, “adopts” has been revised as “used was”.
   31) In page 7 line 154, “adopts” has been revised as “used was”.
   32) In page 8 line 159, to delete “to code in this section”
   33) In page 8 line 160, “it only needs” has been revised as “we only need”.
   34) In page 8 line 161, “At the same time, the CMOS” has been revised as “The CMOS”.
   35) In page 8 line 163, to add “and” between $I_{\text{c}2}(x,y)$, and the.
   36) In page 8 line 165, to delete “coded in this section”.
   37) In page 8 line 165, to delete “Next, the chromaticity imbalance coefficient is used in the bicolor 2+1 3D measurement experiment.”.
   38) In page 8 line 167, “through” has been revised as “by the”.
   39) In page 9 line 175, “,” has been revised as “.”.
   40) In page 14 line 243-244 “Then, to calculate the corresponding root mean square value and make a table1 expressed below:” has been revised as “Then, we calculate the corresponding root mean square errors and made a table as seen in Table 1.”.
   41) In Table 1, “Propose” has been revised as “Proposed method”.
   42) In page 14 line 246, “effect” has been revised as “error”.
