# Supplementary material for: Real-time profilometry by bicolor grating video projection
Source: PLoS One. 2021 Nov 29;16(11):e0259569. doi: 10.1371/journal.pone.0259569 (PMC8629212; doi:10.1371/journal.pone.0259569)
Supplement: Attachment — Submitted filename: Response to Reviewers(minor revision).docx [file pone.0259569.s007.docx]

Dear Editor and Reviewers:

Thank you very much for your excellent editing work of our manuscript entitled “Real-time profilometry by bicolor grating video projection ([PONE-D-21-03909R2] - [EMID:aa41d53618b9d2b1])”. And we also appreciate the reviewers for their valuable comments about our manuscript. Those comments are all valuable and very helpful for revising and improving our manuscript. We have studied the comments carefully and have made the correction which we hope to be able to meet the approval. The Response to Reviewer and Editor Comments is attached below. Hope our revised manuscript meets the high publication standard set by PLOS ONE.

Sincerely,

Yujiao Zhang, Yiping Cao, Haitao Wu, Haihua An, Xiuzhang Huang Sichuan University

Reviewer 4:

1. The label ‘A’ in Fig 3(c) and the label ‘B’ in Fig 5(a) should be moved to their correct positions.

Response:

I'm sorry for making a low-level formatting error. I have changed the format of the two pictures as required.

2. For some sentences, the authors need to pay attention to the usage of tense, such as ‘were’ in line 64 should be ‘are’ and ‘was’ in line 65 should be ‘is’.

Response:

Thank you for checking carefully, I have modified as required.

1) In line 63, “were” has been revised as “are”.
2) In line 64, “was” has been revised as “is”.
3. ‘ ZHANG S' s algorithm’ in both Fig.3 and Fig.5 should be ‘ ZHANG S' algorithm’.

Response:

Thank you for your careful inspection. I have checked the keyword in full and modified it.

1) In line 218, “ZHANG S method.” has been revised as “ZHANG S' method”.
2) In line 232, “ZHANG's method” has been revised as “ZHANG S' method”
